# Depression and glycaemic control among type 2 diabetes patients: a cross-sectional study in a tertiary healthcare facility in Ghana

**DOI:** 10.1186/s12888-018-1933-2

**Published:** 2018-11-06

**Authors:** Josephine Akpalu, Ernest Yorke, Joana Ainuson-Quampah, Williams Balogun, Kwame Yeboah

**Affiliations:** 1 Department of Medicine & Therapeutics, School of Medicine & Dentistry, University of Ghana, Accra, Ghana; 2 Department of Dietetics, School of Biomedical and Allied Health Sciences, University of Ghana, Accra, Ghana; 3 College of Medicine, University of Ibadan, Ibadan, Nigeria; 4 Department of Physiology, School of Biomedical and Allied Health Sciences, University of Ghana, Accra, Ghana

**Keywords:** Depression, Type 2 diabetes mellitus, Glycaemic control, Ghana

## Abstract

**Background:**

Diabetes and depression are both chronic debilitating conditions, and their coexistence has been associated with adverse outcomes. In this study, we investigated the association between glycaemic control and depression in type 2 diabetes (T2DM) patients attending a tertiary healthcare facility in Ghana.

**Methodology:**

In a cross-sectional study design, Patient Health Questionnare-9 (PHQ-9) was used to assess depression in 400 T2DM, aged 30–65 years. Anthropometric characteristics and blood pressure were measured. Venous blood was collected to measure the levels of glycated haemoglobin (HbA1c).

**Results:**

The prevalence of depression was 31.3% among T2DM patients. Female gender, being unmarried, frequent intake of alcohol, previous smoking status and insulin use were associated with increased odds of depression, whereas being educated above basic school level was associated with a decreased odds of depression. In a multivariable logistic regression model, being unmarried and poor glycaemic control were associated with an increase in odds of depression after adjusting for age, gender, and social factors. The association between depression and glycaemic control was attenuated when clinical factors were introduced into the model.

**Conclusion:**

In our study population, we found that depression is common among Ghanaians with T2DM, and not associated with poor glycaemic control in a fully multivariable-adjusted model.

## Background

The prevalence of diabetes mellitus has reached epidemic levels globally resulting in enormous human, economic and social cost worldwide. Currently, 415 million people are living with diabetes, 75% of whom live in low and middle-income countries; this number has been projected to increase to 642 million by 2040 [1]. Depression is also a chronic disease that affects about 340 million people at any given time worldwide [2]. The temporal relationship between diabetes and depression has been found to be bi-directional; patients with depression have an increased risk of 1.6 fold of developing diabetes, whereas diabetes patients are twice as likely to be depressed, compared to non-diabetes individuals [3–5].

The presence of depressive symptoms may affect T2DM patient’s adherence to diabetes self-care regimen, particularly diabetic medications, dietary modifications and exercise [6]. Compared to non-depressed T2DM patients, depressed T2DM patients have relatively higher glycated haemoglobin (HbA1c) and increased prevalence of microvascular and macrovascular complications [7–9]. Screening and treatment of depression in diabetes patients have been reported to favourably improve glycemic control, as well as prevent or delay diabetes-related complications [4]. However, the recognition and treatment of depression among patients with diabetes have been found to be less than optimum [10]. Moreover, in low and middle-income countries including Ghana, there is a dearth of data on the burden of depression among diabetes patients [11, 12].

In this study, we investigated the association between glycaemic control and depression among T2DM patients attending a tertiary care facility in Ghana. We hypothesized that compared to non-depressed diabetes patients, diabetes patients with depression will have poor glycaemic control.

## Methodology

The study was a cross-sectional design, conducted at the National Diabetes Management and Research Centre, Korle Bu Teaching Hospital, Accra, Ghana. The minimum sample size was calculated based on the hypothetical prevalence rates of depression and glycaemic control to be 26% and 15% [13] respectively; 340 patients were required to achieve a power of 80% and α of 0.05. In all, 400 T2DM patients, aged between 30 and 65 years, were enrolled into the study by systematic random sampling of every fourth consenting patient visiting the clinic. Patients with type 1 diabetes, pregnant women and those aged less than 30 years at diagnosis or patients older than 65 years were excluded from the study. In addition, patients with other causes of depression such as loss of a close family member within past 4 months and medication/history of depression or manic/hypomanic episode were excluded from the study. The socio-demographic characteristics such as age, gender, education, employment, alcohol & smoking status, as well as clinical data like duration of diabetes, diabetes medication, hypertension and antihypertensive medication were collected using structured questionnaires.

Depression was screened using Patient Health Questionnaire-9 (PHQ-9). The PHQ-9 is an instrument that has been validated in sub-Saharan Africans [14, 15] and in diabetes patients [16] for the detection of general depressive symptoms**.** The PHQ-9 assesses how often the respondent has experienced specific symptoms over the past 2 weeks, assigning values of 0 to 3 points (0 - not at all, 1 - several days, 2 - more than half of the days, 3 - nearly every day) [17]. Major depressive disorder was defined as the presence of at least five symptoms, reported for more than half the days in the past 2 weeks, including depressed mood or anhedonia, as well as the thought of suicide or better dead [18].

Blood pressure was measured after 5 min rest using an automated digital blood pressure monitor (Omron 907XL pro, Healthcare, Inc., Vernon Hills, IL), with the patients seated comfortably with a back support and arm resting on a table. Body weight and height were measured with a Seca 740 scale and a stadiometer respectively, and the body mass index (BMI) computed using the formula: weight in kilogrammes divided by the height in metres squared.

Venous blood was drawn from each participant for the measurement of glycated haemoglobin (HbA1c) using the boronate affinity chromatography method on PDQ Plus HPLC autoanalyzer (Primus Diagnostics, Trinity Biotech, Ireland). Good glycaemic control was defined as HBA1c of less than 7% [19]**.** Ethical approval was sought and obtained from the Ethical and Protocol Review Committee of the College of Health Sciences, University of Ghana (Protocol ID MS-Et/M.6-P4.4/2012–13) and each patient provided written voluntary informed consent after the rationale and procedure of the study were thoroughly explained. Patients found to be depressed we referred to the Psychiatry department for further assessment and possible management.

### Statistical analysis

Data was analysed using SPSS version 18. Data was presented as mean with standard deviation for continuous variables and as proportions for categorical variables. The study subjects were dichotomized based on the presence or absence of depression. Differences between the two groups with regards to their socio-demographic and clinical variables as well as HbA1c were assessed. The chi-squared (χ^2^) test was used for the comparison of categorical variables and Students t-test for continuous measures. Logistic regression models were performed to determine the change in odds of depression and glycaemic control after adjusting for confounding variables. The level of significance was set at *p* < 0.05.

## Results

In our study population with a high representation of females (male: female = 1:4), 125 (31.3%) of the participants were found to have depression. Age, duration of diabetes and BMI were comparable for T2DM patients with or without depression (T-test, *p*-values> 0.05). Female gender, marital status, educational level, alcohol intake and smoking status were associated with depression (χ^2^, *p*-values< 0.05). T2DM patients with depression had higher systolic blood pressure and glycated haemoglobin level than T2DM patients without depression (T-test, *p* < 0.05; Table 1).

**Table 1:** General characteristics of participants by depression status

	T2DM without depression	T2DM with depression	All Patients	Test statistic	*P*
N (%)	275 (68.7)	125 (31.3)	400		
Age	52.9 ± 8.5	52.2 ± 9	52.7 ± 8.7	t (0.79)	0.48
Duration of diabetes, yrs	9.1 ± 7.3	9.5 ± 6.3	9.2 ± 7	t (0.56)	0.57
Females, n (%)	208 (75.6)	106 (84.6)	314 (78.5)	Χ^2^ (6.72)	0.01
Married n (%)	190 (69.1)	72 (57.6)	262 (65.5)	Χ^2^ (5.02)	0.02
Employed n (%)	194 (70.5)	83 (66.4)	277 (69.2)	Χ^2^ (0.69)	0.41
Education				Χ^2^ (5.04)	0.02
Primary or less	68 (24.7)	41 (32.8)	109 (27.3)		
Junior grade	119 (43.3)	55 (44)	174 (43.5)		
High school	50 (18.2)	19 (15.2)	69 (17.3)		
Tertiary	38 (13.8)	10 (8)	48 (12)		
Alcohol consumption				Χ^2^ (4.86)	0.035
Doesn’t drink	212 (77.1)	92 (73.6)	304 (76)		
Occasional	61 (22.2)	21 (16.8)	90 (22.5)		
Always	2 (0.7)	3 (2.4)	5 (1.3)		
Smoking status				Χ^2^ (5.48)	0.04
Never	260 (94.5)	116 (92.8)	376 (94)		
Previous	14 (5.1)	8 (6.4)	22 (5.5)		
Current	1 (0.4)	1 (0.8)	2 (0.5)		
BMI (mean ± SD)	30.2 ± 6.6	29.4 ± 5.6	29.9 ± 6.3	t (1.18)	0.24
Obesity classification				Χ^2^ (3.99)	0.076
Normal	56 (20.4)	24 (19.2)	80 (20.1)		
Overweight	89 (32.4)	41 (32.8)	130 (32.7)		
Obese	128 (46.5)	60 (48)	188 (47.2)		
Waist circumferece	99.9 ± 13	99.2 ± 11.7	99.7 ± 12.6	t (0.54)	0.58
WHR	0.93 ± 0.11	0.94 ± 0.09	0.94 ± 0.1	t (0.96)	0.34
Hypertension n (%)	219 (79.6)	99 (79.2)	318 (79.5)		0.39
Systolic BP, mmHg	126 ± 26	133 ± 21	132 ± 23	t (2.16)	0.03
Diastolic BP, mmHg	81 ± 12	80 ± 12	81 ± 12	t (0.77)	0.44
Heart rate, bpm	80 ± 15	81 ± 12	80 ± 14	t (0.71)	0.48
FPG, mol/l	9.2 ± 5.5	9.9 ± 4.3	9.4 ± 5.1	t (1.38)	0.17
HbA1c, %	9.4 ± 2.8	10.2 ± 3	9.9 ± 2.9	t (2.52)	0.013

*WHR* waist-hip ratio, *BMI* body mass index, *BP* blood pressure, *FPG* fasting plasma glucose, *HbA1c* glycated haemoglobin, *t* T-test statistic, *Χ*^*2*^ chi-square statistic

In unadjusted logistic regression models, being female (OR = 2.84), unmarried (OR = 1.63), regular alcohol consumer (OR = 5.47), former smoker (OR = 1.28), using insulin (OR = 1.3) and having poor glycaemic control (OR = 1.82) were associated with an increased odds of depression (Table 2). Multivariable logistic regression models were constructed to analyze the relationship between depression versus glycaemic control status and marital status, with sequential adjustments for social and clinical factors. Poor glycaemic control was associated with an increase in odds of depression, even after adjustment for age and gender (AOR = 1.4, *p* = 0.037), as well as social factors (AOR = 1.25, *p* = 0.047); the association was however not significant after the introduction of clinical factors into the model (AOR = 1.04, *p* = 0.286). Being unmarried was associated with increased odds of depression in the fully adjusted model (AOR = 1.47, *p* = 0.046; Table 3).

**Table 2:** Unadjusted logistic regression of depression with socio-demographic and clinical factors

	OR (95% CI)	Wald’s	*p*
Female gender (Ref: Males)	2.84 (1.67–4.07)	4.59	< 0.001
Age (per 1 year change)	1.303 (0.84–1.68)	1.5	0.13
Duration of diabetes ≥10 years (Ref < 10 yrs)	1.05 (0.81–1.46)	0.32	0.76
Unmarried (Ref: Married)	1.63 (1.05–2.54)	2.17	0.003
Above basic school education (Ref: Basic school)	0.64 (0.4–0.93)	2.07	0.037
Alcohol status (Ref: Never)			
Occasional	1.1 (0.66–1.74)	0.39	0.711
Always	5.47 (1.56–13.03)	3.14	0.002
Former Smokers (Ref: Non-smokers)	1.28 (1.05–3.84)	0.75	0.041
BMI (ref: Nornal)			
Overweight	1.07 (0.55–2.1)	0.2	0.851
Obese	0.9 (0.47–1.76)	0.3	0.761
Hypertension (ref: Non-hypertensive)	1.13 (0.87–1.85)	0.63	0.536
Insulin use (Ref: Oral Hypoglycaemic drugs)	1.3 (1.18–1.62)	3.24	0.001
Poor glycaemic control (Ref: HbA1c < 7%)	1.82 (1.32–2.48)	3.72	< 0.001

**Table 3:** Multivariable regression of depression with marital status, medication adherence and glycaemic control

	Unmarried	Wald’s (p)	Poor glycaemic control	Wald’s (p)
Model 1	1.63 (1.15–2.54)	2.42 (0.016)	1.82 (1.32–2.48)	3.72 (< 0.001)
Model 2	1.54 (1.1–2.41)	2.16 (0.031)	1.4 (1.27–2.39)	2.09 (0.037)
Model 3	1.48 (1.09–2.33)	2.02 (0.042)	1.25 (1.17–2.04)	1.98 (0.047)
Model 4	1.46 (1.12–2.31)	2.05 (0.04)	1.04 (0.85–2.29)	0.56 (0.286)
Model 5	1.47 (1.11–2.37)	1.99 (0.046)	–	

This Table represents multivariable logistic regression analyses with depression status as dependent variable, and either marital status, medication adherence or glycaemic control as an independent variable in separate models (Model 1). Further adjustments to the models were performed by introducing variables sequentially as indicated below:

Model 1: Unadjusted

Model 2: Model 1 + age & gender

Model 3: Model 2 + social factors (education, employment, alcohol & smoking status)

Model 4: Model 3 + clinical factors (duration of diabetes, diabetes medication & hypertension)

Model 5: Model 4 + Glycaemic control

## Discussion

Our study has shown that depression is present in about one-third of T2DM patients after screening, and depression was associated with poor glycaemic control. Patients with diabetes have twice the odds of developing depression compared with those without diabetes [4, 5]**.** Depression in T2DM patients has been studied extensively in populations from high-income countries, however, data from lower-income countries such as Ghana are sparse [11, 12].

The prevalence of depression among T2DM patients in our study was similar to that reported in other studies. In a meta-analysis of 42 studies involving over 21,000 adult patients, clinically significant depression was diagnosed in 31% of T2DM patients [5]. Comparable results have also been reported among adult patients with diabetes in Greece (33.4%) [20], rural Bangladesh (30%) [21] and in the UK (25%) [22]**.** In contrast to our findings, relatively lower prevalence of depression was found in studies from rural Pakistan (14.7%) [23] and Brazil (18.6%) [24], with a much lower rate in the United States (8.3%) [25]. On the other hand, using different depression assessment tools, higher rates of depression were reported in urban centers in Iran (71.8%) and Pakistan (43.5%) [11, 26]. The wide variation in depression across various studies may be attributed to the difference in the socio-cultural background of the participants. In screening for depression in the various these studies, different assessment tools with varying sensitivity and specificity to detect depression were used [16].

Very few studies in Africa have evaluated the occurrence of depression and its effects among patients with diabetes [12]**.** However available studies from Nigeria reported the prevalence of depression among T2DM patients within the range of 19.4% to 30%. [27–29]**.** Depression in the general population in Ghana was reported to be rare decades ago, [30] however, in recent years the prevalence has been shown to be comparable to that in western countries [30]. The WHO reported a population-based prevalence of mild depression to be 6.7% among Ghanaian adults > 50 years of age, far lower than the findings of the current study [31]**.** Indeed different studies have reported the prevalence of depression in different Ghanaian populations to be within the range of 3.8 to 9.9% [31–33], and whereas 24.5% of patients referred to a psychiatry clinic were diagnosed with depression [34]**.**

In general, the co-existence of diabetes and depression worldwide has been shown to vary by type of diabetes and the socio-economic status of populations studied [35]**.** Depression and other psychological problems tend to be more common in developing countries compared with developed countries [36–38]. Possible explanations for this observation include higher level of gender inequalities, social insecurity, lower level of education, greater level of poverty and financial constraints among others [37, 38]. Female gender, unmarried status and a lower level of education, all of which have socio-economic implications, were associated with depression in this study. These results are supported by similar findings in both the general population [37, 38] as well as among people with diabetes [22, 39].

Contrary to various study reports, we did not find significant association between poor glycaemic control and depression. Some studies have reported that depressed T2DM patients have higher HbA1c levels compared with non-depressed individuals [25, 40, 41]. The coexistence of depression with diabetes has been reported to be significantly associated with poor glycaemic control [42]. However, this association has not been demonstrated in all studies [43]**.** In systematic reviews and meta-analysis, depression was found to be weakly correlated with glycaemic status [44]. In type 1 diabetes patients, the association between depression and glycaemic control exist in younger patients, and not in adult patients [45]. The findings of our study indicate that, clinical factors like diabetes duration, medication and hypertension status are important factors that link depression to glycaemic status. It must be noted also that predictors of depression in non-diabetes population in Ghana has not been well studied. However, it is worth noting that there is evidence that remission of depression can result in the improvement of glycaemic control [7]. In addition, depression is said to impair good self-care practices resulting in poor adherence to medication, as well as other diabetes management regimens [46]. Diabetes patients with comorbid depression have been found to have fewer days adherence to dietary, exercise and self-monitoring recommendations, with a 2.3 fold increased odds of missing medication doses compared with those without depression [47]**.**

The findings of this study indicate that, compared to unmarried participants, currently married participants had lower risk prevalence of depression. Studies have shown marriage is strongly and positively associated with psychological well-being for men and women. On average, the currently married report higher levels of psychological well-being (measured by lower rates of depression, substance abuse, and alcoholism) than never-married, divorced, widowed, or separated individuals [48, 49]. Spousal support is among the important sources of psychological fortitude during chronic illness like diabetes, although there is a possibility of disruptions in the marital relationship during chronic illness [48]. The importance of marital support in diabetes management might stem from the other spouse helping the ailing spouse to adhere to diabetes medication and self-management practices. Even with married patients, Katz [50] reported that the self-management behavior of husbands with diabetes often deteriorates when conflict exists with their wives. In addition, the belief of a spouse in the importance of glycemic control predicts such control better than the patient’s beliefs [49]. However, in this study, we did not investigate in the quality of intimacy in married participants, as well as the beliefs of the spouse on diabetes management practices.

### Limitation of the study

The major limitation of our study is that the results may not reflect the true burden of depression in the general T2DM population since patients were selected from a specialized tertiary hospital in urban Ghana. Possible replication of this study in a primary health care setting in suburban or rural communities to confirm these findings will be vital. Our study did not include non-diabetes controls for comparison, limiting the interpretation of the possible role of diabetes in depression. Also, being a cross-sectional study by design, we cannot deduce the ‘cause-effect’ relationship between diabetes and depression from our findings; a longitudinal study design will be needed to investigate this relationship.

## Conclusions

The findings of our study have shown that depression is quite common among Ghanaian patients with T2DM. Poor glycaemic control was not associated with depression among T2DM patients in our study. Marriage was found to be protective against depression. Future studies may investigate the role of quality of marital intimacy and spousal beliefs on depression.

## Abbreviations

HbA1c: Glycated haemoglobin
MMAS: Morisky Medication Adherence Scale
PHQ-9: Patient Health Questionnaire-9
T2DM: Type 2 diabetes mellitus
WHO: World Health Organization
